# PTGS1 Regulates Macrophage Polarization Through PPARγ Nuclear Translocation to Affect Oxidative Stress Injury in Chronic Prostatitis

**DOI:** 10.1111/jcmm.71397

**Published:** 2026-10-30

**Authors:** Jian Song, Weiyi Li, Shanchuan Han, Weijie Song, Chaozhao Liang, Yi Liu, Kaiping Zhang, Xianguo Chen

**Affiliations:** ^1^ Department of Urology The First Affiliated Hospital of Anhui Medical University Hefei China; ^2^ Department of Urology The First Affiliated Hospital of Anhui Medical University, Institute of Urology, and Anhui Province Key Laboratory of Urological and Andrological Diseases Research and Medical Transformation Hefei China; ^3^ Department of Urology Anhui Provincial Children's Hospital/Children's Hospital of Fudan University (Affiliated Anhui Branch) Hefei China; ^4^ Department of Urology East City Campus of First Affiliated Hospital of Anhui Medical University Hefei China

**Keywords:** inflammation, macrophage, oxidative stress, prostatitis, PTGS1

## Abstract

Chronic Prostatitis/Chronic Pelvic Pain Syndrome (CP/CPPS) is a condition marked by persistent inflammation of the prostate, whose underlying mechanisms remain poorly defined. This study aimed to investigate the role of prostaglandin‐endoperoxide synthase 1 (PTGS1) in macrophages and in a mouse model of experimental autoimmune prostatitis (EAP). Using multiple experimental methods, we found that PTGS1 was induced during IL‐4‐mediated M2 macrophage polarization, whereas PTGS1 silencing further enhanced M2 polarization. Mechanistically, PTGS1 functioned as a feedback regulatory checkpoint by interacting with PPARγ in the cytoplasm and restraining its nuclear translocation; PTGS1 silencing released this constraint, enhanced PPARγ nuclear accumulation, and promoted M2 macrophage polarization. In EAP mice, PTGS1 knockdown alleviated disease progression and reduced oxidative stress injury, accompanied by increased M2 macrophage polarization and reduced iNOS‐positive pro‐inflammatory macrophage accumulation. Furthermore, PTGS1 silencing diminished the PTGS1‐PPARγ interaction, thereby enhancing PPARγ nuclear translocation and strengthening the M2 polarization program. In summary, our findings show that PTGS1 knockdown attenuates F4/80^+^/iNOS^+^ pro‐inflammatory macrophage accumulation, mitigates prostatic inflammation and oxidative stress, and offers a potential immunomodulatory strategy for CP/CPPS.

## 1 Introduction

CP/CPPS is a common urological disorder that can occur across age groups and is associated with a markedly reduced quality of life. With an estimated lifetime prevalence of 9.3% [1, 2], this condition is characterized by persistent pelvic pain and lower urinary tract symptoms, including urinary difficulty, urgency, and frequency [3, 4]. Because CP/CPPS is multifactorial and often treatment‐resistant, further clarification of its inflammatory and immune mechanisms is urgently needed.

Prostaglandin‐endoperoxide synthase (PTGS/COX) catalyses the committed step in prostaglandin biosynthesis from arachidonic acid and plays an important role in inflammation and immune regulation [5, 6]. PTGS1 and PTGS2 differ in their expression patterns, regulatory mechanisms, and downstream biological functions [5, 7]. Although PTGS1 has traditionally been regarded as a constitutive enzyme involved in homeostatic prostaglandin production, accumulating evidence suggests that PTGS1‐related inflammatory signalling may participate in diverse inflammatory diseases [8, 9].

Immune dysfunction is an important pathological basis for CP/CPPS onset and progression. Among immune cells, macrophages show remarkable plasticity and can polarize into pro‐inflammatory M1‐like or anti‐inflammatory/reparative M2‐like phenotypes [10, 11]. In the CP/CPPS microenvironment, macrophage‐mediated inflammatory remodelling contributes to epithelial injury and disease progression [12, 13]. Within the CP/CPPS microenvironment, M1 macrophages orchestrate inflammatory damage, and macrophage‐mediated alterations to this niche exacerbate epithelial injury and disease progression [14, 15]. However, the molecular mechanisms that regulate macrophage polarization in chronic prostatitis remain incompletely understood.

PPARγ, a member of the nuclear receptor family, is an important regulator of macrophage polarization and inflammatory responses [16, 17]. Activation of PPARγ promotes M2‐like polarization and suppresses pro‐inflammatory signalling [18, 19].

In addition, inflammation‐driven oxidative stress is a key pathological feature of CP/CPPS, in which excessive ROS production and inflammatory cytokine release can form a self‐amplifying cycle of tissue injury [20, 21]. In CP/CPPS, a key pathological feature is the infiltration of diverse immune cells and the sustained expression of pro‐inflammatory cytokines (e.g., IL‐1β, IL‐6, TNF‐α) [22, 23]. This persistent inflammation not only promotes excessive ROS generation but also exacerbates tissue damage and compromises epithelial barrier integrity [24, 25]. Based on these observations, we investigated whether PTGS1 regulates macrophage polarization through PPARγ nuclear translocation and thereby affects inflammation and oxidative stress injury in an EAP mouse model.

## 2 Materials and Methods

### 2.1 Animal Preparation

In this study, male NOD (non‐obese diabetic) mice, aged 5 weeks, were obtained from Nanjing Biomedical Research Centre (Nanjing, China). The Sprague–Dawley (SD) rats were purchased from Hefei Baijin Biotechnology Co. Ltd. and kept in a specific pathogen‐free (SPF) facility under standardized environmental conditions, including controlled temperature, relative humidity, and 12 h light/dark cycle. Each in vivo experimental group contained at least six mice unless otherwise indicated.

### 2.2 EAP Model Establishment

The EAP model was established according to previously published prostate antigen/CFA immunization protocols, with minor modifications. Prostate tissues were collected from SD rats. After homogenizing the prostate tissues, prostate antigen was extracted and combined with complete Freund's adjuvant (CFA) to prepare the immunization reagent. Mice were randomly allocated into five groups: (1) Normal group; (2) EAP group; (3) EAP + sh PTGS1 group; (4) EAP + sh PTGS1 + DMSO group; (5) EAP + sh PTGS1 + GW9662 group. To establish the EAP model, mice were injected into the bilateral axillary and inguinal regions, as well as the ridge section. Immunizations were performed on Days 0 and 14, and the animals were euthanized at the experimental endpoint. Successful EAP induction was confirmed by increased pelvic pain hypersensitivity and histopathological inflammatory infiltration in prostate tissues.

AAV‐sh PTGS1 (200 μL × 10) and AAV‐sh NC (200 μL × 10) were purchased from General Biologicals (Anhui) Co. Ltd. Ten days prior to the first immunization, AAV‐sh PTGS1 (100 μL, titre 1.55 × 10^13^ vg/mL) was injected into the tail vein of mice, while the control group was injected with an equal volume of AAV‐sh NC (titre 1.44 × 10^13^ vg/mL). The sequence used was 5′‐3′ GCAGCACUUGAGUGGCUAUUU. Following AAV infection and the establishment of the EAP mouse model, the EAP group received daily intraperitoneal injections of GW9662 at a concentration of 1 mg/kg, while the control group was treated with DMSO for 14 days before the mice were euthanized.

### 2.3 Behavioural Testing

Twenty‐eight days after the initial immunization, abnormal pain responses were evaluated. Pelvic pain was induced using the von Frey method and monitored in a transparent observation chamber. The responses were classified into three behavioural types: (1) intense abdominal contractions, (2) prompt licking or scratching of the test area, or (3) jumping.

### 2.4 Cell Culture

The immortalized bone marrow‐derived macrophage (iBMDM) cell line was kindly provided by Professor Shao Feng. The cells were cultured in high‐glucose Dulbecco's Modified Eagle Medium (DMEM) supplemented with 10% fetal bovine serum (FBS) and 1% penicillin–streptomycin. Cell culture was maintained in a cell culture incubator at 37°C with 5% CO_2_. To polarize macrophages toward the M2 phenotype, we stimulated iBMDM cells for 24 h with culture medium containing 20 ng/mL IL‐4.

### 2.5 Virus Transfection

Lentiviral transfection was performed in 6‐well plates with approximately 7 × 10^6^ cells per well. After 3 h, the culture medium was replaced, and the cells were transfected with lentivirus at a multiplicity of infection (MOI) of 25. Lentivirus with titre of 1.4 × 10^9^ TU/mL and 4 μL HitransG A was added and the mixture was incubated in a cell incubator for 24 h. After incubation, the supernatant was discarded, and the cells were treated with puromycin (4 μg/mL) for 5 days until no cell death was observed. Sh PTGS1 cells were obtained.

### 2.6 RT‐qPCR

RNA quality was assessed using an instrument, in which absorbance values at 260 nm and 280 nm were measured and their ratio (260/280) was used as an indicator of RNA quality, with values close to 2.0 considered acceptable for downstream applications. The complementary DNA (cDNA) was synthesized by reverse transcription, and a quantitative PCR (qPCR) reaction was prepared using a master mix, specific primers, probes, and cDNA templates. qPCR assays were conducted with the ABI 7500 Real‐Time PCR platform (Applied Biosystems, USA), using SYBR Green chemistry in a 20 μL reaction volume that included cDNA templates, mixtures under specific PCR conditions. The primer sequences used are listed in Table S1.

### 2.7 Western Blotting and Immunoprecipitation

The samples were centrifuged at 13,000 rpm for 10 min under 4 C condition, and the supernatant was collected for subsequent Western blotting experiments. The protein supernatant was mixed with 5 × SDS loading buffer at 4:1 ratio, denatured by heating at 100°C for 10 min. And subjected to electrophoretic separation on a 10% SDS‐polyacrylamide gel under constant voltage. Then the proteins were transferred onto a PVDF membrane. In the end, the membrane was blocked with BSA/TBST solution for 1 h at room temperature. All primary antibody information is listed in Table S2.

iBMDM cells and sh PTGS1 iBMDM cells were seeded into 6‐well plates and stimulated with IL‐4 for 24 h. When the cell density reached 90%, the supernatant was extracted using the above method. One of the wells in the 6‐well plate is reserved as the INPUT sample, while the remaining supernatant was incubated with 3 μg of IgG and the corresponding target protein antibody (anti‐PTGS1). After 12 h, 20 μL of IPKine protein magnetic beads were added to each EP tube. The protein magnetic beads were washed five times with IP lysis buffer and centrifuged at 13,000 rpm at 4°C. Heat the magnetic beads with 50 μL of 5 × SDS loading buffer at 100°C for 10 min.

The target proteins were detected using chemiluminescence. ImageJ software was utilized to measure the grayscale value of proteins.

### 2.8 Flow Cytometry

Prior to flow cytometric analysis, single‐cell suspensions were prepared from mouse spleens or iBMDM cells and washed three times with PBS. The cells were incubated for 1 h at room temperature with 200 μL PBS containing the indicated antibodies. For quantification of macrophages, cells were first gated on the F4/80^+^CD11b^+^ macrophage population. Within this gate, the CD206^+^ population was quantified as M2 macrophages, and the CD86^+^ population was quantified as M1 macrophages. Finally, the cells were washed three more times with PBS before analysis.

### 2.9 RNA Sequencing

Total RNA was isolated from harvested cells using TRIzol reagent, phase separation with chloroform, and RNA precipitation with isopropanol to achieve complete lysis and protect RNA from degradation. RNA quality was assessed before downstream analysis. Purified RNA was used for gene expression analysis. cDNA libraries were prepared and sequenced on the Illumina platform, and differentially expressed genes (DEGs) were identified for downstream enrichment analysis. For the GEO dataset analysis shown in Figure 1A, DEG lists from GSE151015, GSE183074, and GSE208394 were used for Venn diagram analysis, and overlapping genes were selected for subsequent experimental validation. Differentially expressed genes (DEGs) were identified using |log_2_(fold change)| > 1 and *p* < 0.05. The statistical test employed and the screening procedure have been fully described in the updated Materials and Methods section. We believe this addition ensures the reproducibility and transparency of our analysis.

### 2.10 Histopathological Examination

Prostate tissue was preserved in 4% formaldehyde for 24 h, followed by embedding in paraffin and cutting into 5 μm sections. The prostate tissue section was stained with haematoxylin and eosin (H&E) to evaluate the histological findings and visualize the structure and pathological changes of the tissue. The severity of inflammation is classified into four levels, which reflected the extent of histopathological alterations: 0 indicated normal morphology without inflammation; (1), mild perivascular infiltration by mononuclear cells; (2), moderate perivascular infiltration; and (3), marked pathological changes including vascular distortion, haemorrhage, and extensive accumulation of mononuclear cells.

### 2.11 Immunohistochemistry (IHC) Assay

Mouse prostate tissues were collected and fixed in 4% paraformaldehyde (PFA) to preserve tissue morphology and antigen integrity. After fixation, tissue sections were thoroughly washed with PBS. Prior to immunostaining, the sections were permeabilized and blocked following standard protocols. The prostate tissue sections were then incubated with an anti‐4‐HNE antibody, followed by colour development using DAB. Nuclei were counterstained with haematoxylin, and after dehydration and mounting, images were acquired using a digital pathology slide scanner.

### 2.12 Immunofluorescence

All samples were fixed with 4% paraformaldehyde. After fixation, the tissue sections were carefully washed with PBS. The sections were then incubated with PBS containing 0.1%–0.5% Triton X‐100 at room temperature for 10–15 min to increase membrane permeability and enhance antibody penetration efficiency. To minimize background staining caused by non‐specific antibody binding, we blocked the sections with 5% bovine serum albumin (BSA) for 1 h. Finally, the sections were incubated overnight at 4°C with the primary antibody prepared at a specific dilution. Subsequently, the corresponding secondary antibody was added and incubated at room temperature under light‐protected conditions for 2 h. For nuclear staining, the sections were treated with DAPI dye and incubated in the dark at room temperature for 10 min. The sections were then mounted with an anti‐fade mounting medium, and evaluation and image acquisition were conducted using a fluorescence microscope.

### 2.13 ROS Level Measurement

The production of reactive oxygen species in macrophages was quantified with a commercially available detection kit, in which the fluorescent probe becomes oxidized in the presence of ROS, allowing sensitive measurement of intracellular oxidative stress. Before fluorescence was measured, cells were treated with DCFH‐DA (Beyotime, S0034S) at 37°C for 20 min. Afterward, serum‐free culture medium was used three times to wash the cells in order to remove any DCFH‐DA that was not incorporated. ROS levels were assessed using fluorescence microscopy, in which the oxidation of the fluorescent probe generates measurable signals that allow evaluation of intracellular oxidative stress.

### 2.14 Assessment of Oxidative Stress

Malondialdehyde (MDA) is a well‐established biomarker for assessing oxidative stress and lipid peroxidation, while superoxide dismutase (SOD) is a metalloenzyme that neutralizes oxidative free radicals. Serum samples were processed according to the manufacturer's instructions. MDA content was expressed as nmol/mL, and SOD activity was expressed as U/mL. The levels of MDA and SOD in mouse serum were quantified using the MDA assay kit (Beyotime, S0131S) and SOD assay kit (Beyotime, S0101S), respectively, with all experimental procedures performed in strict accordance with the manufacturers' protocols for serum samples.

### 2.15 Determination of Arginase1 Enzyme Activity and Nitric Oxide Production

Cell lysates were normalized using the BCA assay. Arginase activity was quantified with an Arginase Activity Assay Kit (Elabscience, E‐BC‐K484‐M), and NO levels were assessed with a Nitric Oxide Detection Kit (Beyotime, S0023).

### 2.16 TUNEL Assay

The TUNEL assay was applied to evaluate apoptosis by detecting DNA strand breaks in prostate tissue samples. Samples were fixed with 4% paraformaldehyde or a dedicated immunostaining fixative for 30–60 min. After fixation, the sections were incubated with 0.1% Triton X‐100 in PBS for 2 min under 4 C conditions to permeabilize membranes and facilitate probe entry. After that, samples were mounted in antifade medium, visualized under a fluorescence microscope, and apoptotic nuclei were photographed.

### 2.17 Statistical Analysis

All in vitro experiments were repeated no fewer than three times to ensure reproducibility. Every group of mice was composed of at least six animals for in vivo. SPSS version 26.0 software was employed for statistical analysis, whereas Image J was utilized for quantitative image assessment and GraphPad Prism version 9.0 for graph generation. Groups differences were evaluated using Student's *t*‐test or, when appropriate, the Wilcoxon rank‐sum test. A *p* < 0.05 was considered statistically significant.

## 3 Results

### 3.1 PTGS1 Expression Is Altered During IL‐4‐Induced M2 Macrophage Polarization and in EAP‐Associated Inflammatory Tissues

Venn diagram analysis of three GEO datasets related to IL‐4‐induced M2 macrophage polarization (GSE151015, GSE183074, and GSE208394) identified 123 overlapping differentially expressed genes, among which PTGS1 was selected for further validation because of its known involvement in prostaglandin synthesis and inflammatory regulation (Figure 1A and Figure S1). To validate these findings, we treated macrophages with IL‐4 (20 ng/mL, 24 h) to induce M2 polarization and assessed PTGS1 mRNA levels at 4, 12, and 24 h. Quantitative PCR confirmed the upregulation of PTGS1 transcript in M2‐polarized macrophages (Figure 1B). This increase in PTGS1 protein expression during M2 polarization was further corroborated by immunofluorescence (Figure 1C) and western blotting (Figure 1D). Subsequently, we established an experimental autoimmune prostatitis (EAP) mouse model. Double immunofluorescence staining for F4/80 and PTGS1 in prostate tissues from these mice showed reduced PTGS1 expression within infiltrating macrophages (Figure 1E). Consistent with this, western blot analysis of proteins isolated from mouse splenic macrophages (Figure 1F) and prostate tissues (Figure 1G) demonstrated elevated iNOS alongside decreased Arg1 and PTGS1 levels in the EAP group compared to controls. Collectively, these data indicate that PTGS1 is dynamically regulated during IL‐4‐induced M2 polarization and EAP‐associated inflammation, suggesting that PTGS1 may participate in the regulatory network controlling macrophage polarization.

**FIGURE 1:**
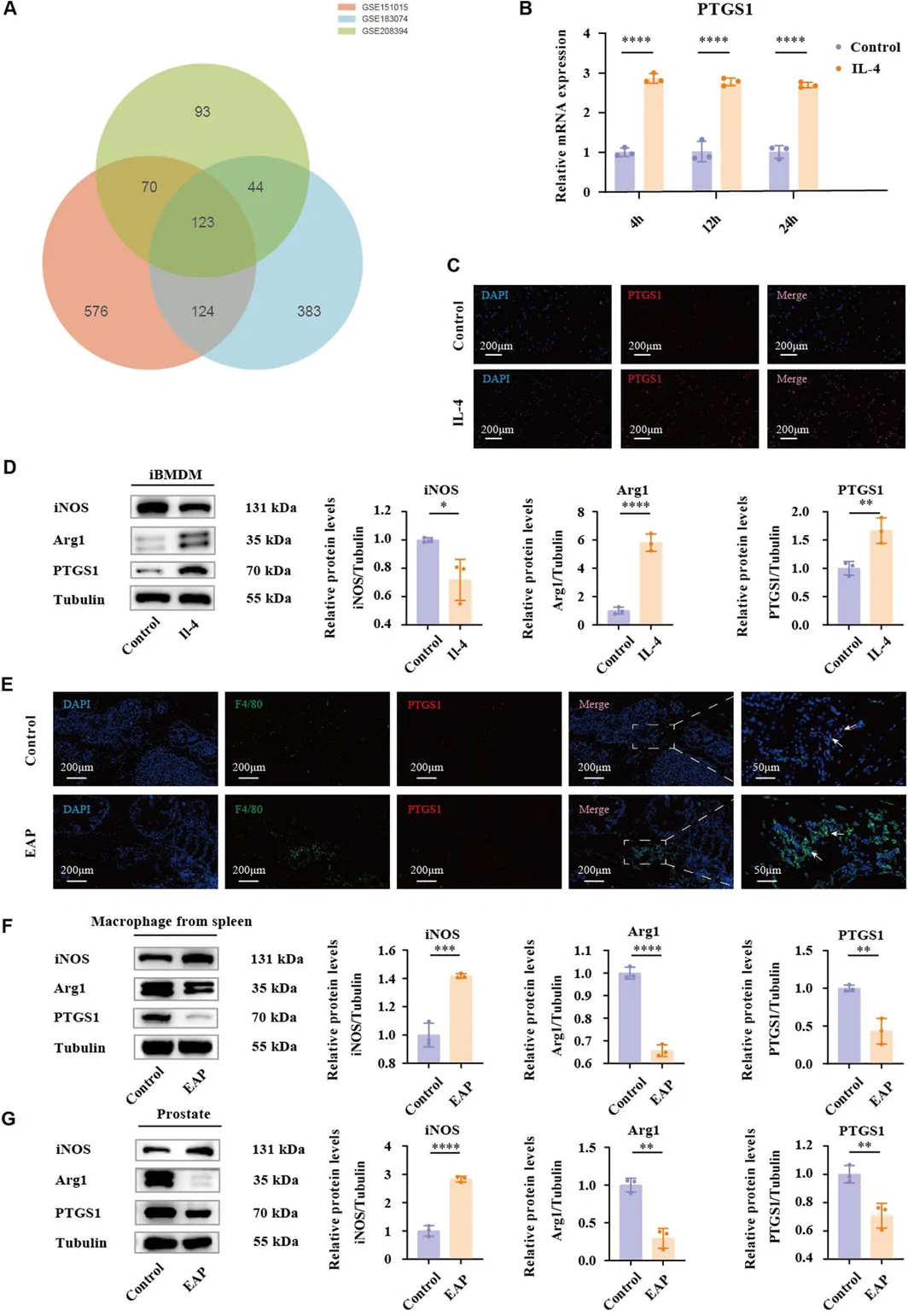
PTGS1 expression is altered during IL‐4‐induced M2 macrophage polarization and in EAP‐associated inflammatory tissues. Venn diagram analysis of three GEO datasets (GSE151015, GSE183074, and GSE208394). (B) RT‐qPCR results of PTGS1 expression at 4 h, 12 h, and 24 h. (C) Immunofluorescence results of PTGS1 in the control group and IL‐4 group. Scale bar = 200 μm. (D) Western blot analysis of iNOS, Arg1, and PTGS1 in control and IL‐4‐treated iBMDMs. (E) Double immunofluorescence staining of F4/80 and PTGS1 in prostate tissues from control and EAP mice. Scale bar = 200 μm. (F‐G) Western blot results of PTGS1, iNOS, and Arg1 in spleen‐derived macrophages and prostate tissue from the control and EAP groups. Data are expressed as mean ± SEM. ns, Not Significant; *p < 0.05; **p < 0.01; ***p < 0.001; ****p < 0.0001.

### 3.2 Silencing PTGS1 Enhances IL‐4‐Induced M2 Macrophage Polarization

We next sought to determine whether PTGS1 directly regulates M2 macrophage polarization. Using lentivirus‐mediated shRNA, we achieved efficient PTGS1 knockdown in macrophages (Figure 2A). Loss of PTGS1 led to a marked induction of M2‐specific markers, as evidenced by western blotting and RT‐qPCR (Figure 2B,C). The upregulation of the M2 marker Arg1 was additionally confirmed by immunofluorescence (Figure 2D). Flow cytometry further showed that PTGS1 depletion increased the CD206^+^ M2 macrophage population under IL‐4 stimulation, indicating that PTGS1 limits the magnitude of IL‐4‐induced M2 polarization (Figure 2E). At the functional level, PTGS1 knockdown augmented arginase activity (Figure 2F) and diminished the secretion of NO and ROS (Figure 2G,H), consistent with an enhanced anti‐inflammatory and reparative capacity. In summary, these findings indicate that PTGS1 acts as a feedback regulatory checkpoint that restrains the full activation of the M2 polarization program.

**FIGURE 2:**
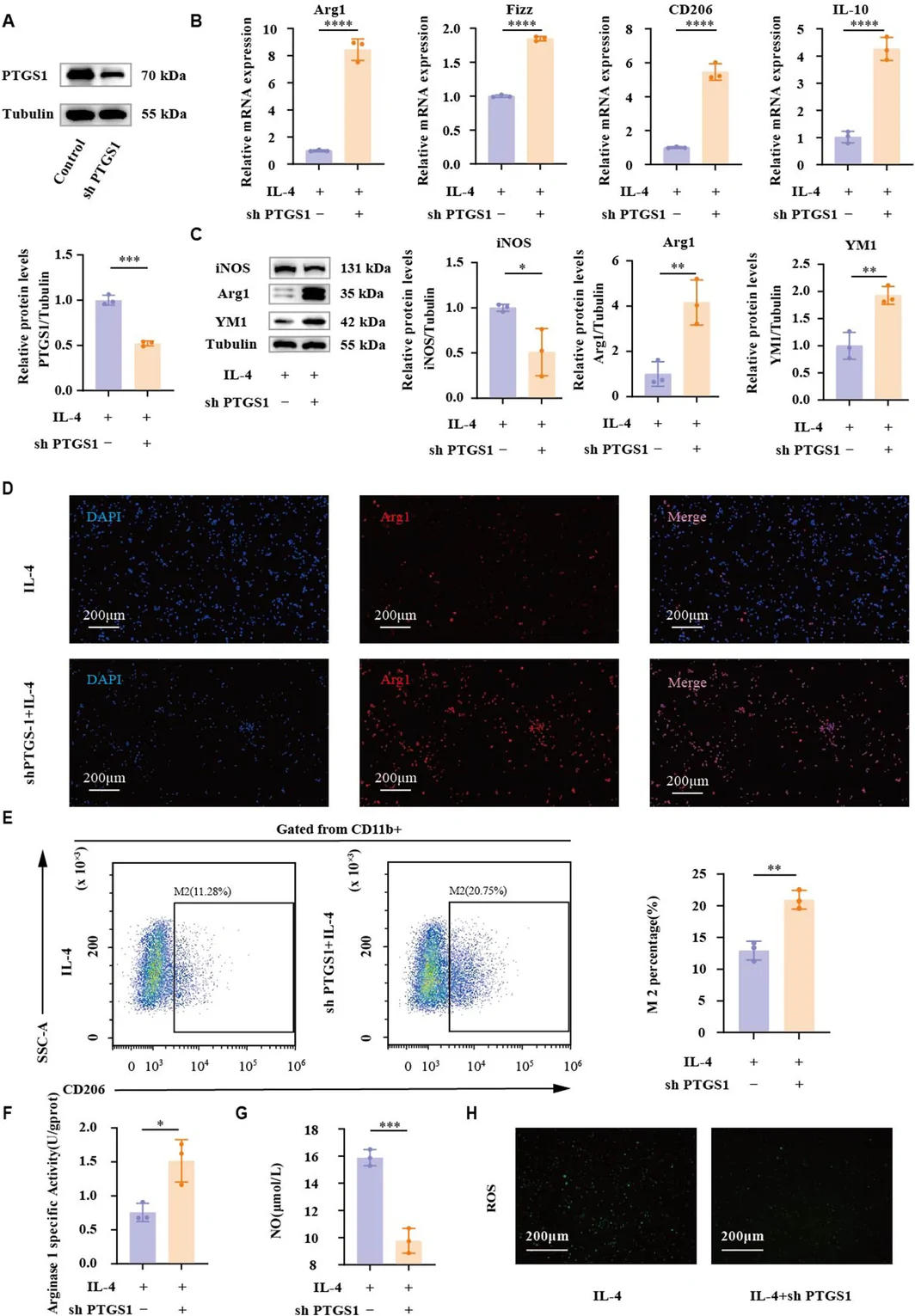
Silencing PTGS1 increases M2 macrophage polarization. (A) Western blot expression of PTGS1 after silencing with lentivirus. (B) Western blot results of iNOS, Arg1, and YM1 in the IL‐4 and IL‐4 + sh PTGS1 groups. (C) RT‐qPCR showing RNA expression levels of Arg1, Fizz, CD206, and IL‐10 in the IL‐4 and IL‐4 + sh PTGS1 groups. (D) Immunofluorescence results of Arg1 in the IL‐4 and IL‐4 + sh PTGS1 groups. Scale bar = 200 μm. (E) Flow cytometry showing the proportion of M2 macrophages in the IL‐4 and IL‐4 + sh PTGS1 groups. (F) NO detection results in the IL‐4 and IL‐4 + sh PTGS1 groups. (G) Arginase activity detection results in the IL‐4 and IL‐4 + sh PTGS1 groups. (H) ROS fluorescence staining results in the IL‐4 and IL‐4 + sh PTGS1 groups. Scale bar = 200 μm. Data are expressed as mean ± SEM. ns, Not Significant; *p < 0.05; **p < 0.01; ***p < 0.001; ****p < 0.0001.

### 3.3 PTGS1 Interacts With PPARγ and Inhibits Its Nuclear Translocation

RNA sequencing of control and PTGS1‐silenced macrophages after IL‐4 stimulation uncovered widespread transcriptomic alterations (Figure 3A). GSEA revealed that PTGS1 knockdown significantly enriched gene sets associated with nuclear receptor signalling (Figure 3B), suggesting its potential role in modulating transcriptional regulators during M2 polarization. To test this hypothesis, we screened for interactions between PTGS1 and several key polarization‐related proteins (P65, TLR4, STAT6, PPARγ) by co‐immunoprecipitation. PPARγ was specifically co‐precipitated with PTGS1 (Figure 3C), and reciprocal Co‐IP validated this interaction (Figure 3D). The binding between PTGS1 and PPARγ was more pronounced in M2 macrophages (Figure 3E). We further demonstrated that PTGS1 knockdown reduced cytoplasmic PPARγ levels and promoted its accumulation in the nucleus (Figure 3F), indicating that the PTGS1‐PPARγ interaction inhibits PPARγ nuclear translocation. These findings demonstrate that PTGS1 interacts with PPARγ and restrains its nuclear translocation, thereby limiting PPARγ‐dependent M2‐promoting transcriptional activity.

**FIGURE 3:**
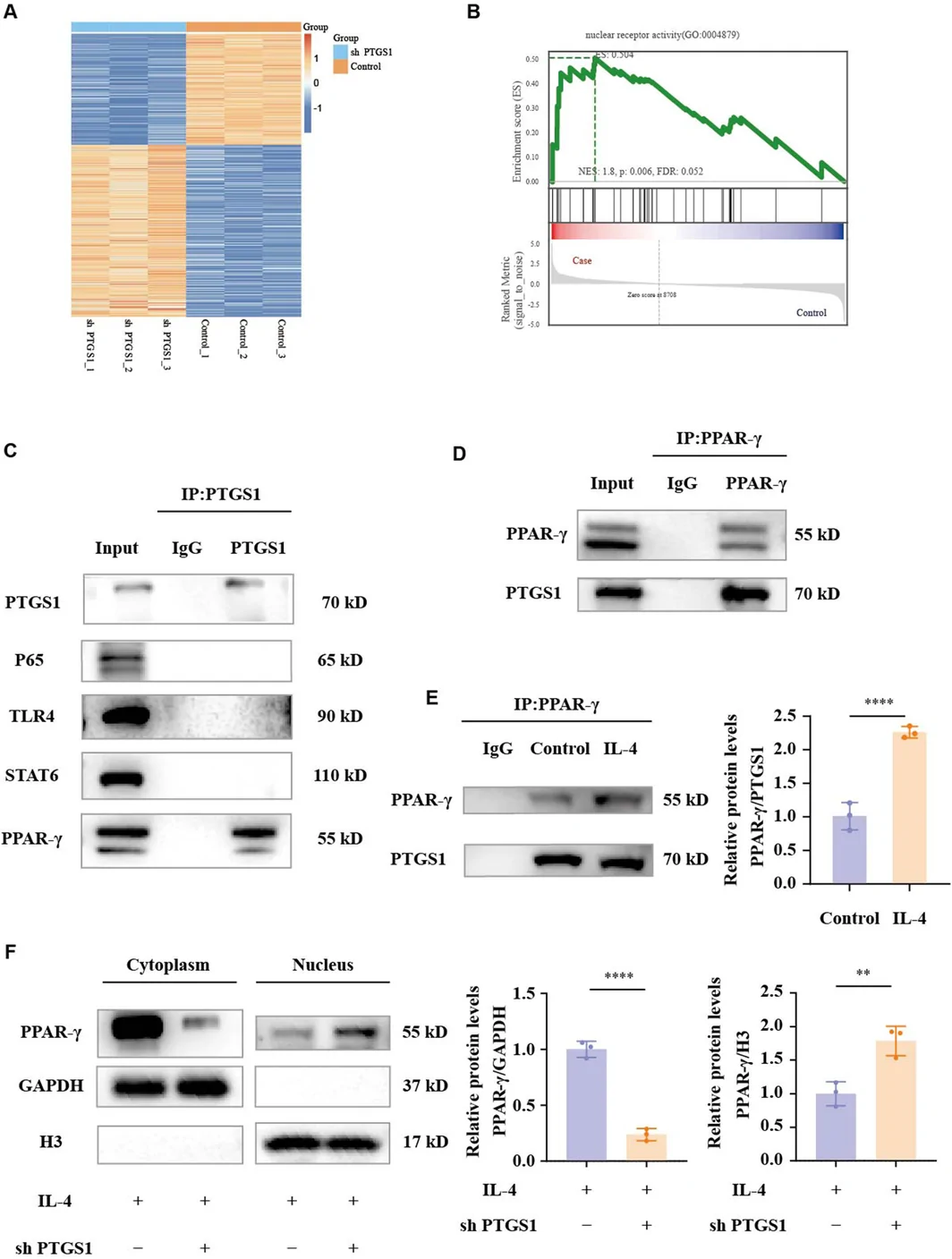
PTGS1 interacts with PPARγ and regulates its nuclear translocation. (A) Heat map of differentially expressed genes following PTGS1 knockdown in iBMDM cells. (B) GSEA following PTGS1 knockdown in iBMDM cells. (C) Immunoprecipitation using anti‐PTGS1 antibody to detect interactions with P65, TLR4, STAT6, and PPARγ. (D) Immunoprecipitation using anti‐PPARγ antibody to detect interactions with PTGS1 and PPARγ. (E) Immunoprecipitation with PTGS1 showing the interaction between PTGS1 and PPARγ in the control and IL‐4 groups. (F) Western blot showing the expression of PPARγ in the cytoplasm and nucleus in the IL‐4 and IL‐4 + sh PTGS1 groups. Data are expressed as mean ± SEM. ns, Not Significant; **p < 0.01; ****p < 0.0001.

### 3.4 PPARγ Inhibitor Reverses the Effects of Lentivirus‐Mediated PTGS1 Silencing on Macrophage M2 Polarization and Cellular Oxidative Stress

To elucidate the dependency of PTGS1‐driven polarization on PPARγ, we assessed the impact of the PPARγ inhibitor GW9662 in PTGS1‐knockdown macrophages. Reversal of the M2 phenotype was observed, as evidenced by the downregulation of characteristic M2 markers and factors (Figure 4A). Western blot analysis indicated that the induction of Arg1 and YM1 following PTGS1 silencing was abolished by GW9662, confirming that their expression is PPARγ‐dependent, unlike iNOS (Figure 4B). Flow cytometry confirmed a significant decrease in the M2 macrophage proportion upon PPARγ inhibition (Figure 4C). Functionally, the anti‐inflammatory and reparative phenotypes—shown by Arg1 expression (Figure 4D) and arginase activity (Figure 4E)—were compromised, while pro‐inflammatory mediators NO and ROS were elevated (Figure 4F,G). These findings conclusively identify PPARγ as the critical mediator through which PTGS1 regulates M2 macrophage polarization.

**FIGURE 4:**
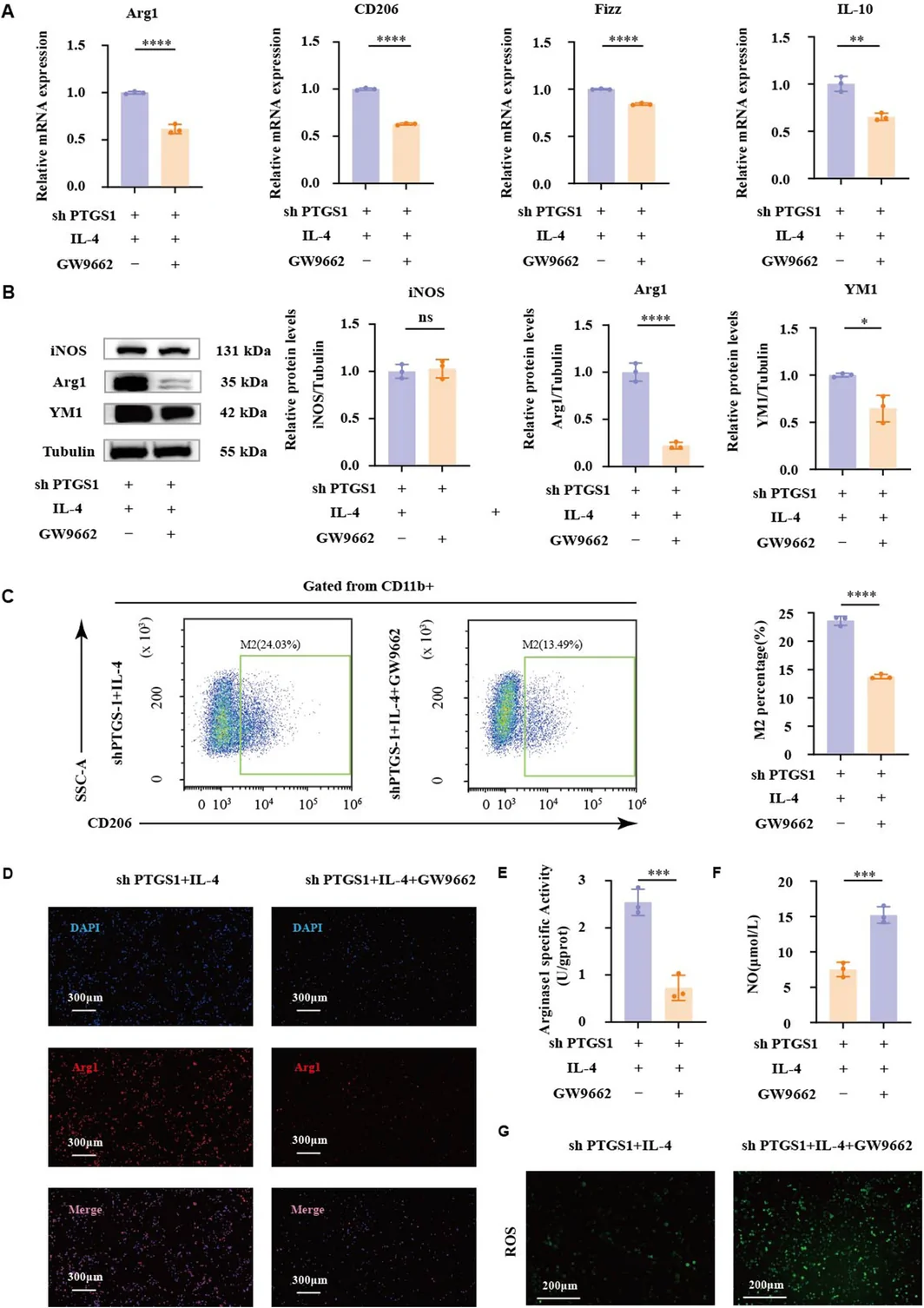
The PPARγ inhibitor reverses the effect of PTGS1 silencing on M2 polarization. (A) Western blot results showing the expression levels of iNOS, Arg1, and YM1 in the IL‐4 + sh PTGS1 and IL‐4 + sh PTGS1 + GW9662 groups. (B) RT‐qPCR results showing the mRNA expression levels of Arg1, Fizz, CD206, and IL‐10 in the IL‐4 + sh PTGS1 and IL‐4 + sh PTGS1 + GW9662 groups. (C) Flow cytometry showing the proportion of M2 macrophages in the IL‐4 + sh PTGS1 and IL‐4 + sh PTGS1 + GW9662 groups. (D) Immunofluorescence staining of Arg1 in the IL‐4 + sh PTGS1 and IL‐4 + sh PTGS1 + GW9662 groups. Scale bar = 300 μm. (E) NO detection results. (F) Arginase activity detection results. (G) ROS fluorescence staining results. Scale bar = 200 μm. Data are expressed as mean ± SEM. ns, Not Significant; *p < 0.05; **p < 0.01; ***p < 0.001; ****p < 0.0001.

### 3.5 PTGS1 Silencing Relieves Inflammation, Increases CD206 ^+^ M2 Macrophages, and Reduces iNOS‐Positive Pro‐Inflammatory Macrophage Accumulation in EAP Mice

A myeloid‐specific PTGS1 knockdown EAP mouse model was successfully constructed (Figure 5A), and the reduction of PTGS1 protein was verified (Figure 5B). Knockdown of PTGS1 led to a significant improvement in disease phenotypes, manifested as a lower frequency of pain responses (Figure 5C) and diminished inflammatory damage in prostate tissue (Figure 5D). At the molecular level, PTGS1 silencing counteracted the EAP‐induced cytokine imbalance by lowering IL‐1β, IL‐6, and TNF‐α and elevating IL‐10 (Figure 5E). To assess the functional consequence on macrophage polarization, we performed flow cytometry and found that PTGS1 knockdown increased the proportion of CD206^+^ M2 macrophages within the F4/80^+^CD11b^+^ macrophage population in the spleen (Figure 5F). In addition, flow cytometry analysis revealed that PTGS1 knockdown also significantly decreased the proportion of M1 (F4/80^+^CD11b^+^CD86^+^) macrophages in the spleen (Figure S2). In prostate tissue, immunofluorescence showed that PTGS1 knockdown reduced F4/80^+^/iNOS^+^ pro‐inflammatory macrophage accumulation (Figure 5G), while CD11b/PTGS1 staining indicated that PTGS1 expression was associated with myeloid cell infiltration in the EAP microenvironment (Figure 5H). These data substantiate that PTGS1 expressed in myeloid cells promotes prostatic inflammation and pain in EAP, at least in part by impairing M2 macrophage polarization.

**FIGURE 5:**
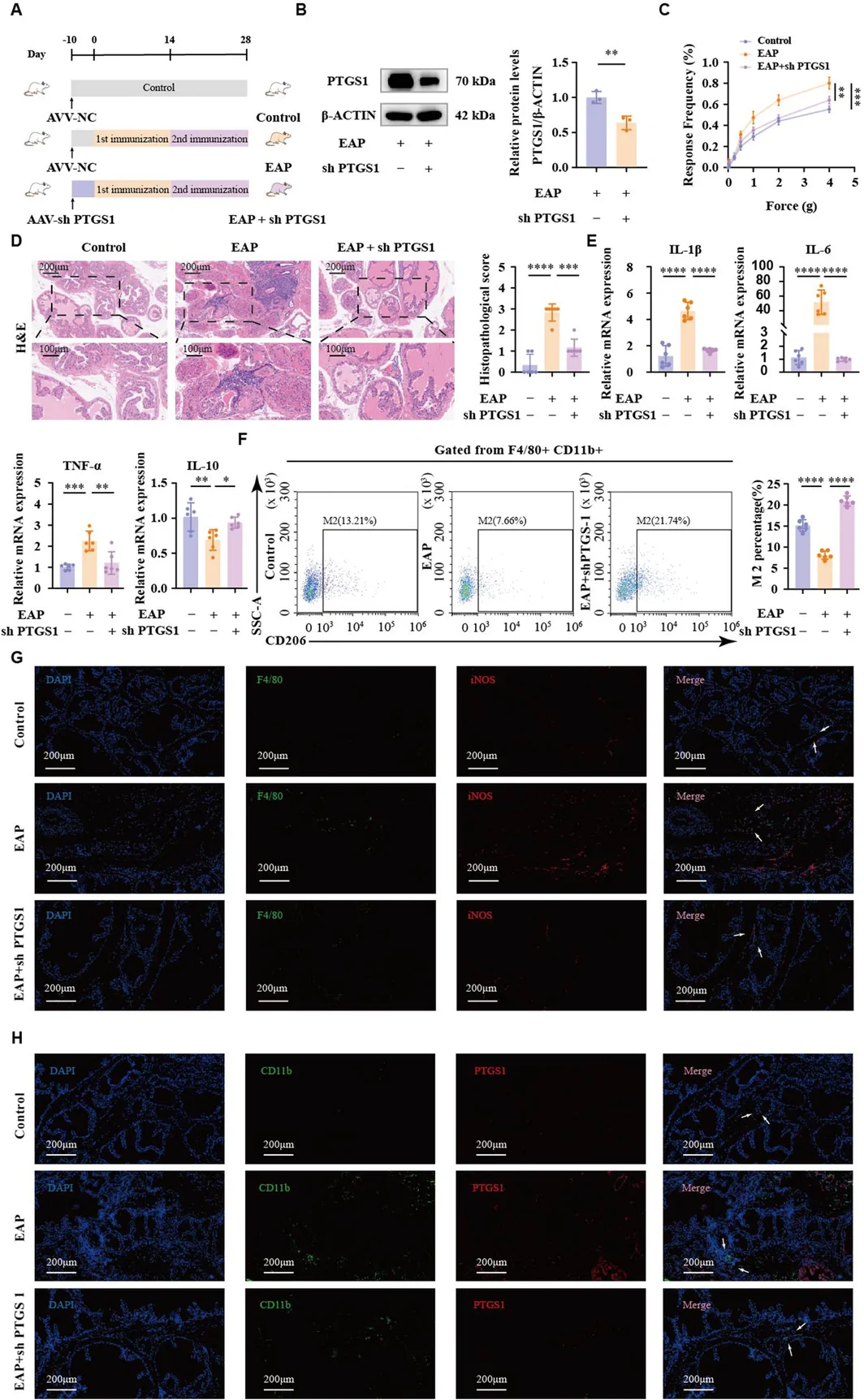
PTGS1 silencing mitigates inflammation, increases CD206+ M2 macrophages, and reduces F4/80+/iNOS+ pro‐inflammatory macrophage accumulation in EAP mice. (A) Schematic diagram of mouse model establishment. (B) Western blot showing the efficiency of PTGS1 silencing by lentivirus. (C) Pain scores of mice in the Control, EAP, and EAP + sh PTGS1 groups. (D) H&E staining results of mouse prostate tissue. Scale bar = 100 μm. (E) RT‐qPCR results showing gene expression levels of IL‐1β, IL‐6, TNF‐α, and IL‐10. (F) Flow cytometry showing the proportion of M2 macrophages in spleen‐derived macrophages. (G‐H) Immunofluorescence double staining results for F4/80, iNOS, CD11b, and PTGS1 in mice prostate tissue. Scale bar = 200 μm. Data are expressed as mean ± SEM. ns, Not Significant; *p < 0.05; **p < 0.01; ***p < 0.001; ****p < 0.0001.

### 3.6 Silencing PTGS1 Relieves Oxidative Stress in EAP Mice

A comprehensive assessment of oxidative stress was performed, evaluating lipid peroxidation (MDA, 4‐HNE), antioxidant capacity (SOD), intracellular ROS, and apoptosis (TUNEL). Analysis of prostate tissues revealed that EAP mice exhibited a significant increase in ROS, which was effectively mitigated by PTGS1 silencing (Figure 6A). Consistent with this, PTGS1 knockdown reduced EAP‐induced cellular apoptosis (Figure 6B) and lipid peroxidation, as indicated by 4‐HNE immunohistochemistry (Figure 6C). Biochemical assays further confirmed that PTGS1 silencing normalized the elevated serum MDA levels and restored serum SOD activity in EAP mice. Given the central role of the NRF2/HO‐1 pathway in combating oxidative stress, we examined its status. Western blot analysis showed upregulation of the NRF2/HO‐1 pathway in EAP mice, which was suppressed upon lentivirus‐mediated PTGS1 knockdown (Figure 6F). These results collectively demonstrate that PTGS1 plays a critical role in mediating oxidative stress damage in the EAP model.

**FIGURE 6:**
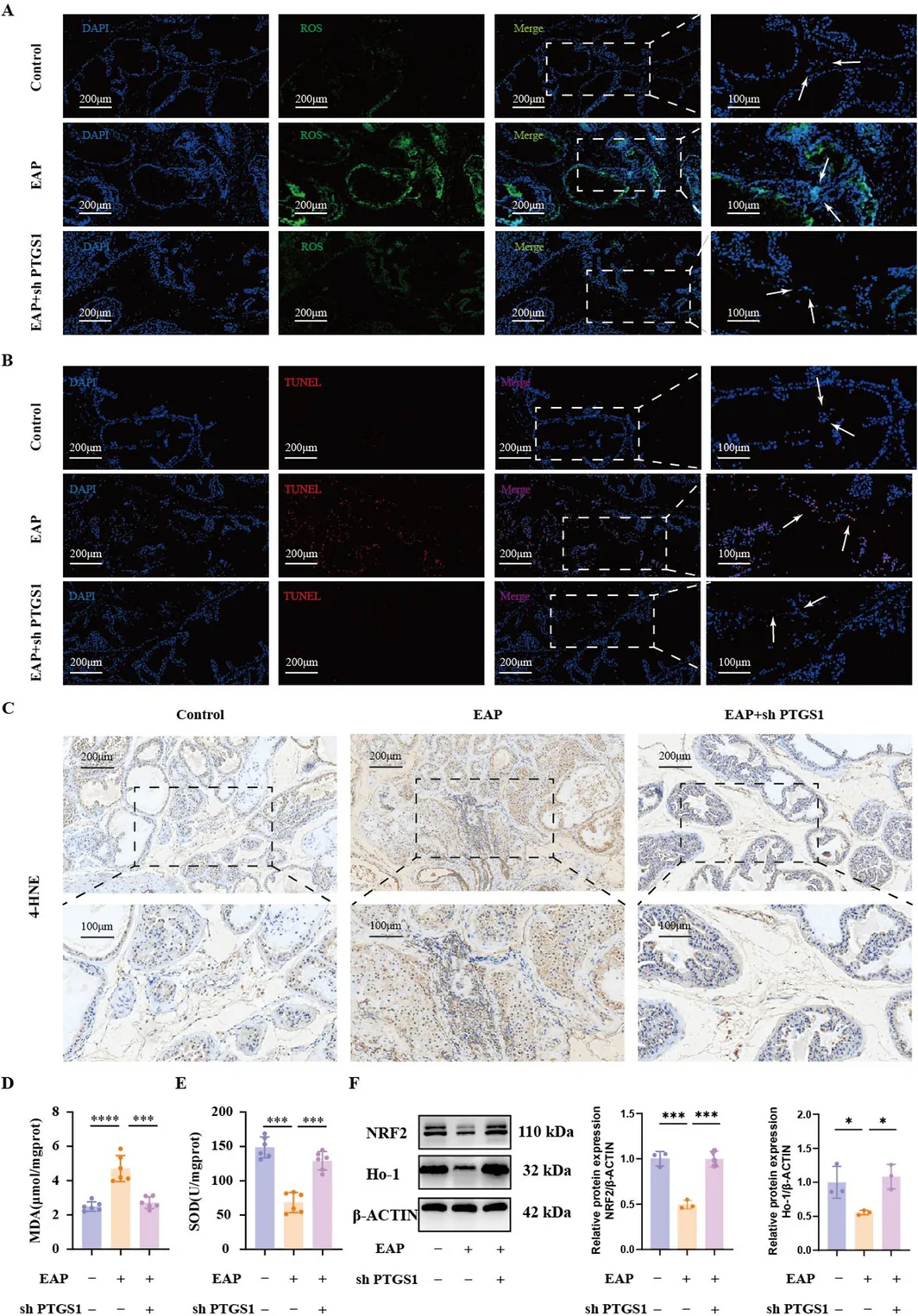
Lentivirus‐mediated silencing of PTGS1 in the EAP model mitigates oxidative stress. (A) ROS staining results in frozen prostate tissue sections from Control, EAP, and EAP + sh PTGS1 groups. Scale bar = 100 μm. (B) TUNEL staining results of mouse prostate tissue. Scale bar = 100 μm. (C) 4‐HNE immunohistochemistry results in mice prostate tissues. Scale bar = 100 μm. (D‐E) MDA and SOD assay results in mice serum. (F) Western blot showing the expression of NRF2/HO‐1. Data are expressed as mean ± SEM. ns, Not Significant; *p < 0.05; ***p < 0.001; ****p < 0.0001.

### 3.7 PPARγ Inhibitor GW9662 Reverses the Effect of Myeloid‐Specific PTGS1 Silencing on Oxidative Stress and M2 Macrophage Proportions in EAP Mice

To definitively establish whether PTGS1 functions through PPARγ in vivo, we administered the PPARγ inhibitor GW9662 to myeloid‐specific PTGS1 knockdown EAP mice on day 14 post‐modelling (Figure 7A). Histological and behavioural analyses revealed that GW9662 treatment abolished the protective effects of PTGS1 silencing, exacerbating prostate tissue inflammation (Figure 7B) and increasing the frequency of pain responses (Figure 7C). Consistently, the cytokine profile shifted toward a pro‐inflammatory state, with elevated levels of IL‐1β, IL‐6, and TNF‐α and a reduction in IL‐10 (Figure 7D). Flow cytometry of splenic cells indicated that the increase in CD206+ M2 macrophages conferred by PTGS1 knockdown was reversed upon PPARγ inhibition (Figure 7E). Furthermore, GW9662 treatment intensified pro‐inflammatory macrophage accumulation and oxidative stress, as evidenced by increased F4/80^+^/iNOS^+^ macrophage signals (Figure 7F), enhanced ROS and TUNEL staining (Figure 7G,H), increased 4‐HNE immunoreactivity (Figure 7I), elevated serum MDA content, and decreased serum SOD activity (Figure 7J,K). The Nrf2/Ho‐1 antioxidant pathway, activated in EAP, was also suppressed by GW9662 (Figure 7L). Collectively, these results confirm that the beneficial effects of PTGS1 silencing on oxidative stress, inflammation, and pain in EAP mice are mediated primarily through the PPARγ pathway.

**FIGURE 7:**
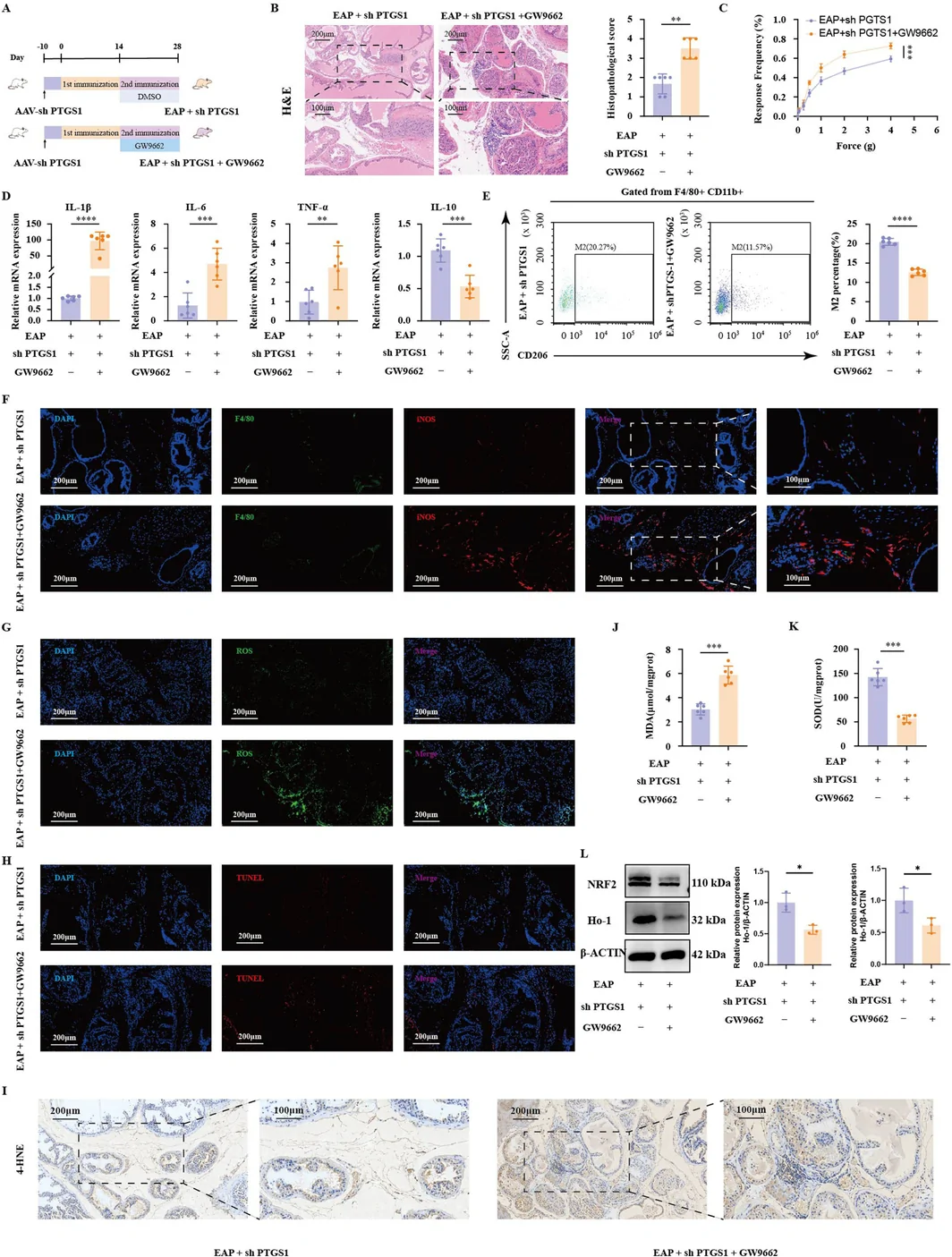
The PPARγ inhibitor reverses the alleviation of inflammation and oxidative stress damage by PTGS1 silencing in EAP mice. (A) Schematic diagram of mouse model establishment. (B) H&E staining results of mice prostate tissues in the EAP + sh PTGS1 and EAP + sh PTGS1 + GW9662 groups. Scale bar = 100 μm. (C) Pain score results of mice. (D) RT‐qPCR showing gene expression levels of IL‐1β, IL‐6, TNF‐α, and IL‐10. (E) Flow cytometry showing the proportion of M2 macrophages in spleen‐derived macrophages in both groups. (F) Immunofluorescence double staining results for F4/80 and iNOS in mice prostate tissues. Scale bar = 100 μm. (G‐H) ROS and TUNEL staining demonstrating changes in oxidative stress levels in mice prostate tissues. Scale bar = 200 μm. (I) 4‐HNE immunohistochemistry results in mice prostate tissues. Scale bar = 100 μm. (J‐K) Serum MDA and SOD assay results. (L) Western blot showing the expression of NRF2/HO‐1 in the groups. Data are expressed as mean ± SEM. ns, Not Significant; *p < 0.05; **p < 0.01; ***p < 0.001; ****p < 0.0001.

## 4 Discussion

A key pathological hallmark of CP/CPPS is the infiltration of diverse immune cells into the prostate tissue. Within this inflammatory microenvironment, sustained release of pro‐inflammatory cytokines—such as IL‐1β, IL‐6, and TNF‐α—not only perpetuates local inflammation but also promotes excessive ROS production. This cascade subsequently aggravates tissue injury and disrupts the integrity of the epithelial barrier [18, 26]. In the present study, we identified PTGS1 as a significant contributor to macrophage‐mediated inflammatory responses and ROS generation, a finding consistently supported by both in vitro experiments in macrophages and in vivo evaluations in the EAP mouse model. Mechanistically, silencing PTGS1 attenuated M1‐like pro‐inflammatory macrophage‐associated signals while facilitating PPARγ nuclear translocation, thereby mitigating inflammatory progression and oxidative stress damage in EAP mice.

M1‐like macrophages contribute to the pro‐inflammatory microenvironment of CP/CPPS by producing inflammatory mediators, including IL‐1β, IL‐6, TNF‐α, NO, and ROS [27, 28]. In the present EAP model, PTGS1 knockdown reduced pro‐inflammatory cytokine expression and decreased F4/80+/iNOS+ macrophage accumulation in prostate tissues, supporting a shift away from a pro‐inflammatory macrophage‐dominant state toward a reparative macrophage phenotype.

PTGS2 has been extensively implicated in the pathogenesis of various inflammatory conditions, where its inhibition has been shown to attenuate inflammatory mediator release and alleviate disease progression—as demonstrated in myocardial infarction [29]. In contrast, the role of PTGS1 in inflammatory processes remains considerably less explored, often being perceived as secondary or complementary to the functions of PTGS2 [30, 31]. Beyond its recognized role in maintaining physiological functions in macrophages, our findings suggest that PTGS1 also plays a pivotal role in macrophage polarization. Prostaglandins (PGs), which act as central mediators of inflammation, are synthesized in a PTGS1‐dependent manner [32, 33]. In the present study, we observed that PTGS1 expression was significantly altered during M2 polarization, with IL‐4 stimulation leading to its marked upregulation. This result not only underscores a previously underappreciated function of PTGS1 in M2 macrophages but also unveils a novel mechanistic insight into its role in inflammatory regulation [34]. An important point clarified by the present study is that IL‐4‐induced PTGS1 upregulation does not necessarily indicate that PTGS1 acts as a positive driver of M2 polarization. Instead, our loss‐of‐function Co‐IP, nuclear fractionation, and GW9662 rescue experiments support a feedback regulatory model in which PTGS1 is induced during IL‐4‐mediated macrophage polarization and subsequently restrains excessive PPARγ nuclear translocation by interacting with PPARγ in the cytoplasm. Thus, PTGS1 knockdown removes this regulatory checkpoint, facilitates PPARγ nuclear accumulation, and strengthens the M2 polarization program. This model reconciles the apparent discrepancy between increased PTGS1 expression during IL‐4‐induced M2 polarization and enhanced M2 polarization after PTGS1 silencing.

Macrophages are pivotal regulators of both the initiation and resolution of inflammatory processes, with their functional plasticity allowing them to adopt opposing phenotypes through polarization into distinct subtypes [35, 36]. In our previous work, we identified a physical interaction between PTGS1 and PPARγ within macrophages and demonstrated that this association modulates PPARγ activity. Specifically, silencing PTGS1 reduced PTGS1‐PPARγ binding, thereby enhancing the nuclear translocation of PPARγ. Given the well‐established role of PPARγ activation in suppressing NF‐κB signalling and attenuating inflammatory responses [37, 38], the upregulation of nuclear PPARγ following PTGS1 knockdown led to a reduction in pro‐inflammatory cytokine release (e.g., IL‐1β, IL‐6, TNF‐α) and alleviated oxidative stress in macrophages.

Infiltration of macrophages represents an early pathological event in CP/CPPS, where excessive ROS production sustains and amplifies inflammatory signalling, creating a self‐perpetuating cycle of tissue injury [39]. In this context, damaged prostate epithelial cells and activated immune cells release large quantities of ROS, which not only fail to resolve inflammation but further aggravate prostate tissue damage, establishing a vicious feedback loop of inflammation and oxidative stress [40, 41]. Our findings indicate that PTGS1 modulates this circuit by promoting macrophage polarization toward the M2 phenotype. Through this mechanism, PTGS1 silencing attenuates inflammatory progression in the EAP model and ultimately mitigates prostatic inflammation and oxidative damage.

From a translational perspective, our study provides a mechanistic rationale for targeting macrophage polarization in CP/CPPS. Current CP/CPPS management remains largely symptom‐oriented, including alpha‐blockers, anti‐inflammatory agents, antibiotics in selected patients, neuromodulatory agents, phytotherapy, pelvic floor physical therapy, and multimodal treatment. However, mechanism‐directed immunomodulatory strategies remain limited. By identifying PTGS1 as a feedback regulatory checkpoint of PPARγ‐dependent macrophage polarization, our study provides a potential therapeutic entry point for developing macrophage‐targeted interventions in CP/CPPS. It should be noted that the precise molecular details by which PTGS1 regulates PPARγ trafficking and activity remain incompletely elucidated and require further mechanistic exploration. In addition, PTGS1 expression was not resolved at single‐cell resolution within the prostatic immune microenvironment. Future studies using single‐cell RNA sequencing, ideally combined with spatial validation, are needed to characterize PTGS1 expression across M1‐like and M2‐like macrophage subsets and to refine the cellular map of PTGS1‐associated immune regulation in prostatitis. Nevertheless, our data clearly underscore the critical role of PTGS1 in the pathogenesis of chronic prostatitis, highlighting its potential as a target for immunomodulatory intervention.

## 5 Conclusions

Our findings demonstrate that PTGS1 acts as a feedback regulatory checkpoint during macrophage polarization. PTGS1 knockdown disrupts the PTGS1‐PPARγ cytoplasmic interaction, thereby facilitating PPARγ nuclear translocation, enhancing M2 macrophage polarization, attenuating F4/80^+^/iNOS^+^ pro‐inflammatory macrophage accumulation, and alleviating inflammation and oxidative stress injury in EAP mice (Figure 8). These results identify the PTGS1‐PPARγ axis as a potential immunomodulatory target for CP/CPPS.

**FIGURE 8:**
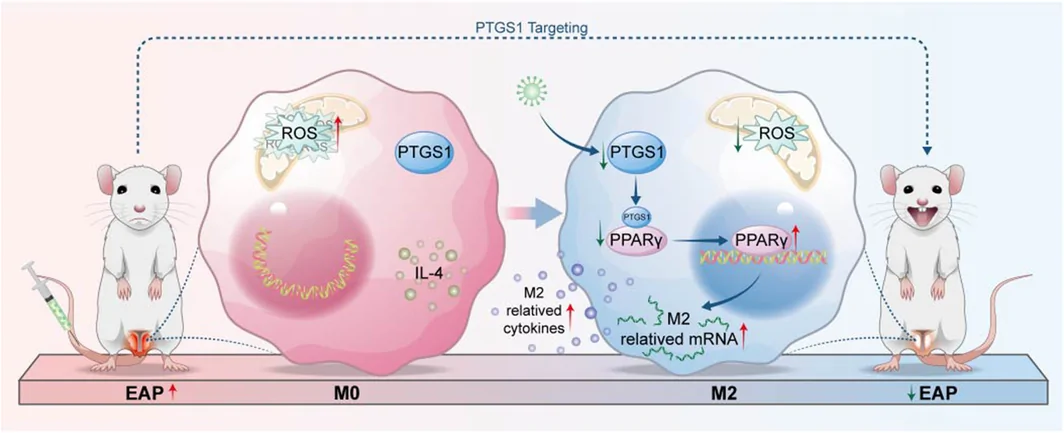
Mechanistic diagram of PTGS1 influencing macrophage polarization on CP/CPPS progression. Silencing the PTGS1 gene reduces the binding between PTGS1 protein and PPARγ protein in the cytoplasm, leading to increased translocation of PPARγ into the nucleus. This enhances its transcriptional activity, promotes M2 polarization of macrophages, and alleviates oxidative stress injury.

## Author Contributions

**Jian Song:** writing – original draft, writing – review and editing, conceptualization. **Weiyi Li:** formal analysis, project administration. **Shanchuan Han:** investigation, data curation. **Weijie Song:** formal analysis, visualization. **Chaozhao Liang:** supervision. **Yi Liu:** resources. **Kaiping Zhang:** project administration, software, validation. **Xianguo Chen:** methodology, funding acquisition.

## Funding

This work was funded by the National Natural Science Foundation of China (grant no. 9021340202).

## Ethics Statement

The Committee for Animal Care and Use of the Animal Centre of Anhui Medical University approved all the animal protocols.

## Conflicts of Interest

The authors declare no conflicts of interest.

## Supporting information


**Figure S1:** Relative PTGS1 expression in M0 versus M2 macrophages across three GEO datasets. (A)The bar plot shows the relative expression levels of PTGS1 in M2 macrophages compared with M0 macrophages (set to 1.0) in three independent GEO GSE151015, GSE183074, and GSE208394. Expression values were normalized to the M0 group for each dataset. PTGS1 was significantly upregulated in M2 macrophages relative to M0 across all three datasets (all *p* < 0.05). Data are expressed as mean ± SEM. ns, Not Significant; *p < 0.05; **p < 0.01; ***p < 0.001; ****p < 0.0001.

**Figure S2:** Flow cytometry analysis of M1 macrophage populations in spleen‐derived macrophages.

**Table S1:** Primer sequences used for RT‐qPCR.

**Table S2:** Antibodies and kits used in Western blotting, flow cytometry, and functional assays.

## Data Availability

The data that support the findings of this study are openly available in SRA (Sequence Read Archive) at https://www.ncbi.nlm.nih.gov/bioproject/PRJNA1335419, reference number PRJNA1335419.
