# Supplementary material for: PTGS1 Regulates Macrophage Polarization Through PPARγ Nuclear Translocation to Affect Oxidative Stress Injury in Chronic Prostatitis
Source: J Cell Mol Med. 2026 Oct 30;30(21):e71397. doi: 10.1111/jcmm.71397 (PMC13721901; doi:10.1111/jcmm.71397)
Supplement: Supplementary file 1 [file JCMM-30-e71397-s002.docx]

**Fig. S1. Relative PTGS1 expression in M0 versus M2 macrophages across three GEO datasets.**
(A)The bar plot shows the relative expression levels of PTGS1 in M2 macrophages compared with M0 macrophages (set to 1.0) in three independent GEO datasets: GSE151015, GSE183074, and GSE208394. Expression values were normalized to the M0 group for each dataset. PTGS1 was significantly upregulated in M2 macrophages relative to M0 across all three datasets (all * *p* * < 0.05). Data are expressed as mean ± SEM. ns, Not Significant; **p* < 0.05; ***p* < 0.01; ****p* < 0.001; *****p* < 0.0001.
