# Supplementary material for: PTGS1 Regulates Macrophage Polarization Through PPARγ Nuclear Translocation to Affect Oxidative Stress Injury in Chronic Prostatitis
Source: J Cell Mol Med. 2026 Oct 30;30(21):e71397. doi: 10.1111/jcmm.71397 (PMC13721901; doi:10.1111/jcmm.71397)
Supplement: Supplementary file 2 [file JCMM-30-e71397-s004.docx]

**Figure S2. Flow cytometry analysis of M1 macrophage populations in spleen-derived macrophages.**

(A) Quantitative analysis of the proportion of M1 macrophages (F4/80⁺CD11b⁺CD86⁺) in the Control, EAP, and EAP shPTGS1 groups. Knockdown of PTGS1 significantly reduced the proportion of M1 macrophages compared to the EAP group. Data are expressed as mean ± SEM. ns, Not Significant; **p* < 0.05; ***p* < 0.01; ****p* < 0.001; *****p* < 0.0001.
